# Timing of Urbanisation and Cardiovascular Risks in Thailand: Evidence From 51 936 Members of the Thai Cohort Study, 2005–2009

**DOI:** 10.2188/jea.JE20140063

**Published:** 2014-11-05

**Authors:** Jiaying Zhao, Sam-ang Seubsman, Adrian Sleigh, the Thai Cohort Study Team

**Affiliations:** 1 National Centre for Epidemiology and Population Health, ANU College of Medicine, Biology and Environment, the Australian National University, Canberra, Australia; 2 School of Human Ecology, Sukhothai Thammathirat Open University, Pakkret Nonthaburi, Thailand

**Keywords:** urbanization, recent migrants, cardiovascular disease, Thailand, Thai Cohort Study

## Abstract

**Background:**

Urban populations usually have higher levels of cardiovascular risk factors than rural populations in developing countries. However, association between cardiovascular risk factors and duration of urban dwelling, particularly for early stages of urban migrations, has not yet been adequately studied. We examined cardiovascular risks in relation to timing of urbanization in Thailand, paying attention to recent internal migrants.

**Methods:**

Our study base was a large national cohort (*n* = 87 151) of distance-learning Thai open university students recruited in 2005 and followed up in 2009. After exclusion of longitudinal dropouts and reverse migrants, 51 936 remained for analyses. The information collected included historical residence, urban migration and its lifecycle timing, self-reported doctor-diagnosed diseases, and socio-demographic and personal attributes that could influence health. To relate cardiovascular outcomes (prevalence and incidence of hypertension and hyperlipidaemia) and life-course urbanization status (ie at age 12, 4 years ago [2005] and at present [2009]), we applied logistic regression. Included in the models were 10 other covariates that could confound the urbanization effect.

**Results:**

Recent migration (arriving within four years) among young cohort members (born after 1980) was associated with higher risk of hypertension (OR 1.80 for prevalence and 1.68 for four-year incidence). Higher hyperlipidaemia prevalence (and incidence) was associated with any urban dwelling. Recent migrants quickly developed hyperlipidaemia, particularly the youngest (born after 1980) and oldest participants (born before 1960).

**Conclusions:**

Increased cardiovascular risks appear among rural-urban migrants within four years after they arrive. Given the scale of continuing urbanization, interventions are needed to support and educate recent migrants in Thai cities.

## INTRODUCTION

Urban populations have higher prevalence of cardiovascular risks than rural populations in some developing countries.^1^^–^^3^ Two recent cross-sectional studies explored the association between duration of urban dwelling and cardiovascular risk factors in India.^4^^,^^5^ Both studies suggested that rural-urban migrants have higher prevalence of obesity than rural dwellers. In addition, body fat was found to increase rapidly after moving to an urban environment, while prevalence of other cardiovascular risk factors increased more gradually. However, research on the relationship between timing of urban migration and cardiovascular risk factors (eg, hypertension and hyperlipidaemia) is still limited, especially in developing countries, the worldwide setting of massive urban migration. Information based on longitudinal data is even more limited. An improved understanding of cardiovascular risks after urban migration, especially in the early stages of migration, would improve knowledge of transitions to chronic diseases and the window of opportunity for disease control.^4^

There is extensive literature on migration and health. One review focused on acculturation and health in Asian immigrants, evaluating findings in 67 articles addressing aspects of this topic.^6^ One phenomenon recurs across the literature on migration and health: rural-urban migrants are initially healthier than urban host populations; however, after a lag period, the migrants develop diet and lifestyles that increases risk of chronic diseases, especially cardiovascular disease and diabetes.^6^^,^^7^

The loss of the migrant health advantage was recently investigated in China.^7^ The author noted a deterioration in health behaviour and lifestyle as well as an increase in acculturative stress along with psychological disorders. Urbanization is proceeding intensively in Southeast Asia, and more information is therefore needed on urban migration and health, particularly on emerging cardiovascular disease.^8^

Thailand is a developing country undergoing urbanization, with the proportion of the population living in cities rising from 20.9% in 1970 to 34.1% in 2011.^9^ It is expected that 1.6% of the Thai population will urbanize each year from 2010 to 2015, and this movement involves over one million people annually.^10^ So, over the next 10 to 20 years, around 6% of the population at any one time will have arrived in an urbanized area in the last 4 years. Fortunately, Thailand has introduced a universal health care program, so urban migrants in Thailand have good access to health services, limiting one possible confounder of migration effects.^7^^,^^11^^–^^13^

Information on the link between urbanization and cardiovascular risk factors in Thailand is sparse. However, the ongoing urbanization of the Thai population provides an opportunity to examine the effect of the timing of urbanization on the occurrence of cardiovascular risk factors of hypertension and hyperlipidaemia.

We aim to improve understanding of cardiovascular risks in relation to timing of urbanization in Thailand. We focus on hypertension and hyperlipidaemia and use data from a large community-based nationwide Thai Cohort Study (TCS) to investigate two important urbanization hypotheses related to the health-risk transition. The first hypothesis is that long-term urban dwellers have a higher cardiovascular risk than long-term rural dwellers; the second hypothesis is that cardiovascular risk can increase quickly during the early period of rural-urban migration (within 4 years).

## METHODS

### Source of data

The TCS is a longitudinal study on the health consequences of socioeconomic development.^14^^,^^15^ Participants were distance-learning adult students of Sukhothai Thammathirat Open University (STOU) residing nationwide in 2005; 87 151 responded to the baseline 20-page health risk questionnaire (44% of the student body). A follow-up was conducted in 2009 (*n* = 60 569; response rate >70%).^16^^,^^17^ For this report, we analysed the prospective cohort data covering the period 2005 to 2009.

STOU students attend university to improve their life situation but cannot afford on-campus education. Most therefore remain embedded in their communities, work, and families. The cohort represented STOU students well for age, sex, course of study, social geography, and socioeconomic status.^14^ The cohort also represented average Thais for sex ratio, median age, religion, income, occupation, and geographic location.^14^^,^^15^

### Measurements

We collected information on place of residence at three life-course milestones: at age 10–12 years, 4 years ago (in 2005), and present (in 2009). We chose the age of 10–12 as it comes at the midpoint of childhood and is an age at which reliable memories are formed. The questions in 2005 were: “where was your permanent home when you were a child 10–12 years of age (Countryside or City/Town)” and “where is your current permanent home located now (Countryside or City/Town)”. In 2009, we asked “where is your current residence located (Countryside or City/Town)”. We categorized the timing of urbanization by noting urban (U) or rural (R) status at each of these three milestones (RRR, RRU, RUU, UUU). RRR or UUU is described as long-term rural or urban dwellers, respectively. RRU persons are recent urban migrants who migrated to an urban area during 2005 to 2009. RUU persons are medium-term urban migrants who were in rural areas when they were aged 10–12 years, but had migrated to an urban area by 2005.

We were interested in the timing of rural-to-urban migration and its effect on cardiovascular risk factors. Some reverse migrants may be going to a rural area due to illness or retirement, and they do so against a general trend toward urbanization.^18^ Therefore, we excluded people who moved back to rural areas during 2005–2009. We also excluded those with missing values for life-course milestones. Overall, 51 936 cohort members (86%) were analysed.

Cardiovascular outcomes were self-reported doctor-diagnosed hypertension and hyperlipidaemia. The accuracy of the cohort data on hypertension was investigated in a separate validation study by physician telephone interviews of a cohort sub-sample of 240 hypertensives and 240 normotensives.^19^ The physician used a medical algorithm (place of diagnosis, drug treatment, and blood pressure measurements) to establish a “gold standard” outcome. Self-reported results for hypertension were acceptable (sensitivity 82%, specificity 71%, overall accuracy 75%). The sensitivity reported in the validation study means that 82% of true hypertension cases would be detected by self-report in our cohort. The results of the validation study also revealed that a very high proportion of negative self-reports would be accurate (greater than 98%) due to the high negative predictive value reported in that earlier report.^19^ This high negative accuracy arises in part from a low Bayesian prior probability, given that less than 10 percent of the cohort would be expected to have hypertension. One further point of note in reference to diagnostic error involves the effects of diagnostic error on epidemiologic estimates, such as the odds ratio (OR). Non-differential errors will move the OR towards the null (OR = 1), but ORs that remain significantly different from the null are valid although the true effect is actually larger than that detected.

While we did not perform a parallel investigation of self-reported hyperlipidaemia, we nevertheless expect comparable accuracy, as the diagnosis is not easily confused. Since 2001, all Thais have had access to adequate primary health care, including basic investigations such as serum cholesterol.^20^ Given the high level of education of cohort members and the impact of a universal coverage scheme on rural access to health care, we believe geographically differential under-reporting of hyperlipidaemia would be uncommon.

Potential confounders were studied because they were known or suspected to relate to both the outcomes (hypertension or hyperlipidaemia) and the exposure of interest (urbanization). These included sex, age groups (measured by birth year group [up to 1959, 1960–1969, 1970–1979, and 1980 or later]), family history, income, health service access, the type of health insurance coverage, smoking, drinking, physical activity, and body mass index (BMI). Family history was derived from a question in 2005 regarding the cause of death (diabetes or high blood pressure) if the respondent’s mother or father had died. Monthly personal income in Thai Baht (1 US dollar is equal to 35 Baht) was measured in 2009 and analysed in 4 categories (‘≤7000’, ‘7001–10 000’, ‘10 001–20 000’, or ‘>20 001’). Health service access was measured by the question: “in the past 12 months, have you personally used any of the following health services”. We classified responses into ‘only used public services’, ‘used private services at least once’, and ‘others—not using public or private services’. We also controlled for the type of health insurance coverage, classified as ‘30 Baht scheme’, ‘civil servant benefit scheme’, and ‘others’. Smoking in 2009 was classified as ‘never’, ‘ex-smoker’, or ‘current smoker’. Alcohol consumption in 2009 was classified as ‘non-drinker’, ‘light drinker (≤7 glasses per week)’, or ‘moderate or heavy drinker (≥8 glasses per week)’.

Information on exercise-related weekly physical activity (PA) in 2009 was obtained by asking: “during a typical week (7-day period), how many times on average do you do each of these physical activities: walking continuously for at least 10 minutes; moderate physical activity for more than 20 minutes; or vigorous physical activity for more than 20 minutes”. Metabolically-adjusted exercise-related PA (number of sessions) was calculated by ‘walking + moderate physical activity + 2 × vigorous physical activity’; finally, PA was recoded as ‘<7 sessions’ or ‘≥7 sessions’. This derived measure of PA was based on the International Physical Activity Questionnaire and the Active Australia Survey.^21^ Using self-reported Asian standards, BMI was used to classify respondents as obese (BMI ≥ 25.0), overweight at risk (BMI = 23.0–24.9), normal weight (BMI = 18.5–22.9), and underweight (BMI < 18.5) in 2009.^22^

### Statistical analysis

We used logistic regression to assess urbanization risks for the two cardiovascular outcomes hypertension and hyperlipidaemia. The outcome measures were prevalence in 2009 and incidence from 2005 to 2009. The exposure of interest was urbanization status across life course (RRR, RRU, RUU, UUU). RRR was selected as the reference group. We adjusted effect estimates (ORs) for confounding factors. We also examined multiplicative interactions for urbanization-sex. To provide more control on the influence of age, analyses were repeated after stratifying by birth year (four groups) and within strata, including age in years as a potential confounder within each of the four models. We also used stratified analyses to further investigate interactions with our variable of interest. Stratified birth cohort analyses were conducted separately for hypertension and hyperlipidaemia. For the multivariate logistic regression models, Hosmer-Lemeshow tests were used to determine whether or not models were adequately fitted (*P*-value > 5%). Individuals with missing data were excluded from the multivariate analyses.

### Dropouts

At each stage of analysis, there were baseline cohort members who dropped out. This arose due to loss of contact at the 4-year follow-up (*n* = 26 582), reverse migration (urban to rural) (*n* = 8633), missing variables required for the modelling (*n* = 5217), or initial prevalence at baseline making incidence determination impossible (*n* = 2250 for hypertension and *n* = 4633 for hyperlipidaemia). These dropouts are progressively described in the relevant sub-sections above and are summarized as a flow diagram in Figure. We evaluated the impact of dropouts by comparing the baseline (2005) status for the analysed group to the unanalysed group for all the explanatory variables included in the models. Results revealed minor differences; compared values for each variable were all within 10% of each other, except for birth year. The proportions for the oldest age groups (born before 1960) were similar (5.4% vs 6.8%) but dropouts were more numerous (36.2% vs 25.3%) in the youngest group (born after 1979). However, this was a minor discrepancy, and when all younger cohort members (born after 1959) were combined, proportions of the younger age represented within compared groups were very similar (93.2% of the analysed group and 94.6% of the unanalysed group). Thus, even though dropouts were a little younger on average, the variables of interest in our analyses were not greatly influenced by dropouts. We therefore conclude that the dropouts did not bias our results for the variables we analysed.

**Figure.**
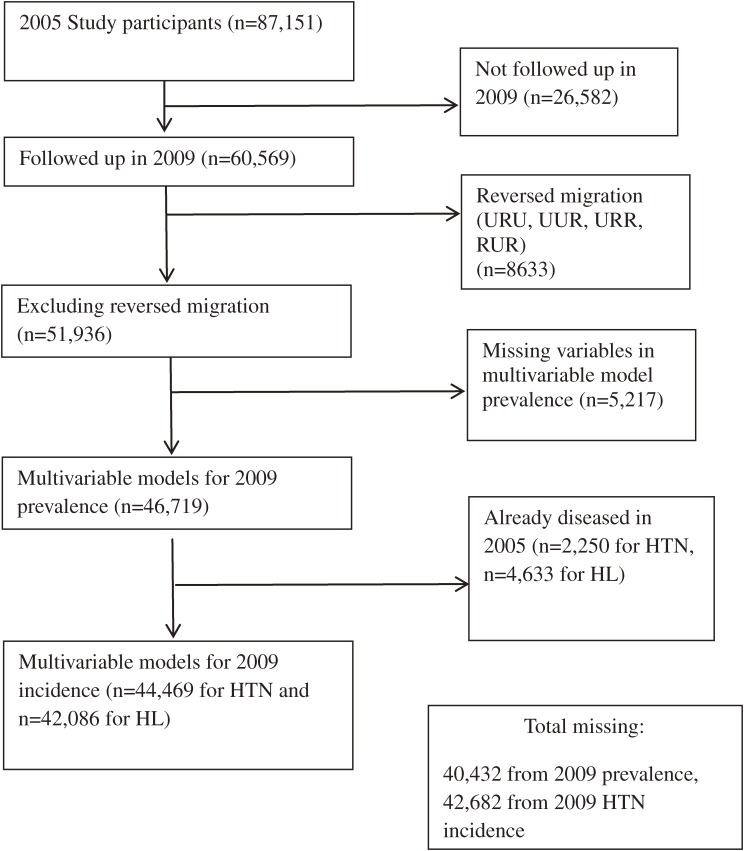
Cohort analyses flow chart showing analysed groups, dropouts, and exclusions. HL, hyperlipidaemia; HTN, hypertension.

## RESULTS

### Demographic characteristics and urbanisation of the cohort members

Overall, 51 936 cohort members had suitable data for our study (23 342 men [45.3%] and 33 162 women [54.7%]) (Table 1). A total of 93% were born after 1959 and were aged 49 years or less in 2009.

**Table 1.** Distribution of major demographic, behavioural, and physical characteristics of cohort members by life-course urbanization status and sex^a^

Cohort member attributes		Totals (%)	Distribution of urbanization categories by sex (%)^b^							

			Males				Females			
	
			RRR	RRU	RUU	UUU	RRR	RRU	RUU	UUU
		*n* = 51 936	(*n* = 9519)	(*n* = 2567)	(*n* = 6837)	(*n* = 4419)	(*n* = 10 725)	(*n* = 3202)	(*n* = 8004)	(*n* = 6663)
Birth year	–1959	7.27	9.31	5.73	12.84	13.69	2.50	2.12	4.70	8.21
	1960–1969	24.14	27.07	22.67	31.4	28.02	18.14	15.24	23.24	25.48
	1970–1979	43.68	44.50	46.28	42.75	40.37	43.29	44.78	47.04	40.70
	1980–	24.92	19.12	25.32	13.00	17.92	36.07	37.85	25.02	25.60

Income (Baht)	≤7000	18.17	23.83	11.96	6.82	13.33	28.90	16.71	14.57	15.13
	7001–10 000	22.05	18.95	20.92	14.33	14.08	31.13	28.98	24.71	18.94
	10 001–20 000	35.15	36.81	42.58	43.26	31.89	25.92	36.66	38.34	34.01
	≥20 001	22.41	18.46	22.79	34.08	38.56	11.47	15.33	19.94	29.19

Health service access	Public services only	37.57	49.57	40.94	36.67	29.85	43.38	33.76	28.84	28.13
	Private services	39.65	26.52	32.92	37.11	41.68	38.18	44.82	49.74	50.01
	Others	22.78	23.91	26.14	26.22	28.47	18.43	21.42	21.43	21.87

Health insurance coverage	30 Baht scheme^c^	12.66	19.29	10.60	4.87	9.44	20.35	12.77	6.41	9.20
	Civil servant scheme	27.68	33.32	32.49	37.19	27.31	23.16	21.99	23.39	23.43
	Others	59.66	47.39	56.91	57.93	63.25	56.48	65.24	70.20	67.37

Smoking	Never	76.05	52.72	55.24	52.07	51.53	96.38	95.94	95.26	92.93
	Ex-smoker	8.51	18.15	17.02	17.22	20.16	0.28	0.47	0.57	1.47
	Current smoker	13.87	27.72	26.18	29.24	27.06	1.43	2.34	2.44	4.13

Alcohol consumption	Non-drinker	69.09	45.27	44.88	46.03	55.80	86.75	87.07	86.36	87.12
	Light drinker	14.88	26.59	25.67	24.35	18.31	7.04	7.21	7.42	7.29
	Moderate/heavy drinker	11.71	23.15	24.78	24.92	22.61	1.38	2.34	1.94	2.42

Weekly physical activity	<7 sessions	16.13	9.99	12.43	12.49	16.41	18.00	20.71	18.33	22.03
	≥7 sessions	81.17	87.17	84.50	83.93	80.92	79.58	76.89	79.01	75.87

BMI	<18.5	9.50	4.29	3.47	2.74	4.12	14.55	16.96	13.78	12.97
	18.5–22.9	49.34	44.06	44.68	39.05	35.96	56.73	56.28	58.06	52.39
	23.0–24.9	18.54	24.08	25.87	25.99	23.85	13.59	12.21	13.82	13.30
	≥25.0	22.62	26.21	25.05	30.91	35.14	14.36	13.65	13.67	20.68

Family history of hypertension	No	94.60	94.60	94.23	94.15	91.72	95.89	96.10	95.39	93.40
	Yes	5.40	5.40	5.77	5.85	8.28	4.11	3.90	4.61	6.60

^a^Based on place of residence at three time markers in the life-course: age 10–12 years, in 2005, and in 2009. Each marker is coded as U (urban) or R (rural).

^b^For each tabulated cohort member attribute, the urbanization-sex categories may not add up to 100% due to missing data.

^c^‘30 Baht scheme’ is the name given to the comprehensive universal health insurance available to Thai citizens since 2001.

Only 18.9% of males and 23.3% of females had lived in urban areas at all three milestones in the life-course (UUU); by 2005, around half the cohort members (48.2% of males and 51.3% of females) had urbanized (derived from Table 1). Over the next four years (2005–2009), another 11.0% of males and 11.2% of females moved from rural to urban areas (RRU). Overall, 40.8% of males and 37.5% of females lived in rural areas at all three measurement points (RRR) of the life-course (aged 10–12 years, in 2005, and in 2009).

### Distribution of potential confounders

Incomes were modest (less than US $700/year for >80%) and were lower for rural residence at all times. Rural people were more likely to only use public health services and to only have the 30 Baht scheme of health insurance. Smoking was not related to urban residence for males. The patterns were different for females with very low smoking rates overall but a threefold increase for UUU residents compared to RRR residents. Alcohol had a very similar pattern, although the female difference between urban and rural was less marked. For both males and females, physical activity was about 10% less for UUU residents than RRR residents. BMI was markedly higher for UUU residents among both sexes. Compared to RRR residents, UUU residents reported a more than 50% higher rate of family history of hypertension.

### Hypertension and hyperlipidaemia

In 2009, the prevalence of hypertension was 6.0%, and the prevalence of hyperlipidaemia was 14.4%. From 2005 to 2009, the four-year incidence of hypertension was 3.4%, and that of hyperlipidaemia was 8.6% (Table 2). Background risks (incidence and prevalence) for both outcomes (hypertension and hyperlipidaemia) increased steadily across birth cohort groups as age increased. Long-term urban dwellers (UUU) had higher risks of prevalent hypertension. There was also a small tendency for long-term urban dwellers to have a higher incidence of hypertension, but, for the two older age groups (born before 1970), there was no evidence of increased hypertension incidence for UUU. Hyperlipidaemia was more straightforward, with higher prevalence and incidence among long-term urban dwellers for all birth cohorts.

**Table 2.** Hypertension and hyperlipidaemia prevalence in 2009 and cumulative incidence from 2005 to 2009^a^

	Birth year				Total

	–1959	1960–1969	1970–1979	1980–	
	Hypertension prevalence (%)				

RRR	21.58	8.93	3.19	1.34	5.00
RRU	23.26	9.53	3.43	2.15	4.89
RUU	23.60	9.33	3.39	1.52	6.34
UUU	25.43	10.42	4.47	2.32	7.74

Total	23.52	9.46	3.53	1.68	5.96

	Hypertension four-year incidence (%)				

RRR	12.12	5.54	2.23	1.04	3.10
RRU	13.14	5.78	1.94	1.59	2.87
RUU	10.72	5.50	2.31	1.10	3.53
UUU	11.71	5.88	2.69	1.87	4.13

Total	11.59	5.63	2.31	1.29	3.42

	Hyperlipidaemia prevalence (%)				

RRR	29.29	22.53	7.22	1.92	10.41
RRU	40.93	28.04	9.27	3.97	12.22
RUU	42.58	30.32	12.28	4.36	18.17
UUU	40.45	28.44	12.32	5.04	17.88

Total	37.77	26.88	9.96	3.36	14.42

	Hyperlipidaemia four-year incidence (%)				

RRR	16.34	13.90	5.66	1.73	6.64
RRU	27.67	14.04	7.33	3.54	7.67
RUU	23.63	17.34	8.79	3.62	10.57
UUU	21.29	16.51	8.51	4.01	10.27

Total	20.71	15.56	7.31	2.85	8.59

^a^Based on 51 936 cohort members with recorded life-course urbanization in one of the analysed categories (UUU, RUU, RRU, or RRR); for life-course codes, see footnote in Table 1.

ORs relating timing of urbanization to hypertension and hyperlipidaemia were calculated for 2009 prevalence (Table 3) and 4-year incidence (Table 4). In each table, the adjusted ORs are presented for the whole cohort as well as for each of the four-birth-year strata. The models all fit adequately (*P*-value > 0.05 in Hosmer-Lemeshow tests). Any multiplicative interactions between urbanization and sex were non-significant.

**Table 3.** Timing of urbanization status of Thai cohort members and prevalence odds ratios for hypertension or hyperlipidaemia in 2009 stratified by birth cohort group^a^

Urbanization status	All		Birth year							

			<1960		1960–1969		1970–1979		1980–	
	(*n* = 46 719)		(*n* = 3186)		(*n* = 11 144)		(*n* = 20 571)		(*n* = 11 818)	
				
	OR	95% CI	OR	95% CI	OR	95% CI	OR	95% CI	OR	95% CI
	Hypertension									

RRR	ref		ref		ref		ref		ref	
RRU	1.22*	1.05–1.41	1.17	0.80–1.72	1.11	0.87–1.43	1.20	0.93–1.56	1.80***	1.19–2.72
RUU	1.06	0.95–1.18	1.04	0.83–1.30	1.01	0.86–1.19	1.06	0.87–1.29	1.23	0.81–1.86
UUU	1.34***	1.20–1.49	1.36**	1.08–1.70	1.21*	1.01–1.45	1.33**	1.08–1.64	1.60***	1.09–2.33
Hosmer-Lemeshow *P*-value	0.13		0.86		0.86		0.77		0.84	

	Hyperlipidaemia									

RRR	ref		ref		ref		ref		ref	
RRU	1.29***	1.43–2.70	1.65***	1.18–2.32	1.24**	1.05–1.47	1.26**	1.07–1.49	1.79***	1.31–2.45
RUU	1.49***	1.60–1.76	1.68***	1.38–2.04	1.35***	1.21–1.51	1.54***	1.36–1.73	1.73***	1.31–2.29
UUU	1.48***	1.60–2.49	1.64***	1.34–2.01	1.34***	1.19–1.51	1.49***	1.30–1.70	2.14***	1.62–2.83
Hosmer-Lemeshow *P*-value	0.36		0.97		0.78		0.13		0.9	

*<0.05, **<0.01, ***<0.005.

^a^All models based on cohort members with complete data; for life-course codes, see footnote in Table 1. All odds ratios were adjusted for the following confounders: sex, income, health service access, quality of health insurance coverage, smoking, drinking, physical activity, and body mass index. Estimates based on whole cohort ORs were also adjusted for birth year groups, and estimates for each of the four birth year strata were adjusted for age in years. Hypertension models were adjusted for family history of hypertension.

**Table 4.** Timing of urbanization status of Thai cohort members and odds ratios for incident hypertension or hyperlipidaemia from 2005 to 2009, stratified by birth cohort group^a^

Urbanization status	All		Birth year							

			<1960		1960–1969		1970–1979		1980–	
				
	OR^b^	95% CI	OR	95% CI	OR	95% CI	OR	95% CI	OR	95% CI
	Hypertension									

*n* = 44 469		*n* = 2619		*n* = 10 377		*n* = 19 866		*n* = 11 607	

RRR	ref		ref		ref		ref		ref	
RRU	1.11	0.92–1.34	1.11	0.66–1.88	1.05	0.76–1.46	1.03	0.74–1.44	1.68*	1.05–2.69
RUU	0.98	0.85–1.12	0.81	0.59–1.11	0.96	0.78–1.20	1.05	0.82–1.33	1.09	0.67–1.76
UUU	1.17*	1.02–1.35	1.02	0.74–1.40	1.11	0.88–1.40	1.15	0.89–1.49	1.61*	1.06–2.46
Hosmer-Lemeshow *P*-value	0.13		0.49		0.32		0.36		0.34	

	Hyperlipidaemia									

*n* = 42 086		*n* = 2141		*n* = 8845		*n* = 19 426		*n* = 11 674	

RRR	ref		ref		ref		ref		ref	
RRU	1.20***	1.06–1.36	1.81**	1.17–2.79	0.91	0.72–1.15	1.31**	1.08–1.58	1.78***	1.31–2.45
RUU	1.34***	1.23–1.47	1.45**	1.10–1.91	1.18*	1.02–1.36	1.43***	1.24–1.64	1.60***	1.31–2.29
UUU	1.34***	1.21–1.47	1.33	1.00–1.78	1.19*	1.02–1.40	1.39***	1.19–1.62	1.94***	1.62–2.83
Hosmer-Lemeshow *P*-value	0.44		0.72		0.06		0.13		0.84	

*<0.05, **<0.01, ***<0.005.

^a^All models based on cohort members with complete data; for life-course codes, see footnote in Table 1. All odds ratios were adjusted for the following confounders: sex, income, health service access, quality of health insurance coverage, smoking, drinking, physical activity, and body mass index. Estimates based on whole cohort ORs were also adjusted for birth year groups, and estimates for each of the four birth year strata were adjusted for age in years. Hypertension models were adjusted for family history of hypertension.

^b^Odds ratio estimating relative cumulative incidence (relative risk).

The actual timing of urbanization between age 12 and 2005 depended on a person’s birth cohort. By stratifying into four birth cohort groups and repeating analyses of urbanization effects within groups, we know that urbanization probably occurred long ago for RUU persons in older groups and more recently in the younger groups.

Hypertension was associated with urbanization. Long-term urban dwellers (UUU) had higher odds of hypertension prevalence in 2009 (OR 1.34, 95% confidence interval [CI] 1.20–1.49). Recent rural-urban migrants (RRU) who had moved to urban areas between 2005 and 2009 were also at greater risk of hypertension (OR 1.22, 95% CI 1.05–1.41), but this increase was not observed among medium-term urban migrants. The OR of incident hypertension from 2005 to 2009 was also significant (OR 1.17, 95% CI 1.02–1.35) for long-term urban dwellers (UUU).

When we further investigated risk stratified by birth cohort groups, prevalence showed a rapid increase in young people (born after 1980) among recent migrants (OR 1.80, 95% CI 1.19–2.72) but not in older people. For incident hypertension, the pattern is the same for young recent migrants (OR 1.68, 95% CI 1.05–2.69).

The prevalence of hyperlipidaemia showed substantial and significant effects of urbanization. Relative to long-term rural dwellers (RRR), any levels of urban dwelling time increased the risk of hyperlipidaemia (ORs from 1.29 to 1.48), even affecting recent migrants (RRU). For incidence of hyperlipidaemia, the trends are similar.

Urbanization effects were modelled within four birth cohort groups. Long-term urban dwellers (UUU) and medium-term urban migrants (RUU) had higher risks of prevalent and incident hyperlipidaemia than long-term rural dwellers (RRR). These risks were increased across all birth cohort groups. Recent migrants (RRU) were quickly affected, showing higher prevalent and incident risks for hyperlipidaemia, particularly for the youngest (born after 1980) and oldest cohorts (born before 1960).

Finally, we tested the two hypotheses mentioned in the Introduction. For hypothesis 1 (H1: *that long term urban dwellers have a higher cardiovascular risk than long-term rural dwellers*), we compared the cardiovascular risk of long-term urban dwellers (UUU) to long-term rural dwellers (RRR). ORs for prevalent hypertension and hyperlipidaemia all support H1, across all age groups (Tables 3 and 4). The ORs for incident hyperlipidaemia across all age groups also support H1. However, the OR of incident hypertension only increased among the youngest age cohort, not among older age groups.

We then tested hypothesis 2 (H2: *that cardiovascular risk can increase quickly during the early stage of rural-urban migration [within 4 years]*) by comparing cardiovascular risk for recent migrants (RRU) with that for long-term rural dwellers (RRR). A rapid increase in risk of hypertension among recent migrants appeared for young people, but not for older people. Hyperlipidaemia risk for new migrants increased significantly across three of the four birth cohorts.

## DISCUSSION

We studied a large sample of Thais living nationwide and analysed 51 936 (45.3% male). Few (21.3%) had lived in urban areas when aged 10–12 years, with most migrating subsequently. From 2005 to 2009, 11.1% moved from rural to urban areas. In 2009, the prevalence of hypertension was 6%, and the prevalence of hyperlipidaemia was 14.4%. The 4-year incidences of hypertension and hyperlipidaemia were 3.4% and 8.6%, respectively. Hypertension was associated with recent migration among young cohorts (OR 1.80 for prevalence and 1.68 for four-year incidence). Hyperlipidaemia prevalence was associated with any urban dwelling across all age groups. For disease incidence, trends were similar. Risks for prevalent and incident hyperlipidaemia increased quickly in recent migrants (RRU), particularly for the youngest and oldest cohorts.

Increased cardiovascular risks have been reported in other Asian countries experiencing rapid urbanization.^23^^,^^24^ Our research in a large cohort of mostly young adult Thais showed that recent migration (within 4 years) increased the risk for hypertension among young cohort members. Any urbanization (recent or not) was associated with hyperlipidaemia.

The increase in risk of hypertension among new migrants for the youngest birth cohort in our study (RRU) is generally consistent with the results in previous studies, suggesting that changes in blood pressure may occur within a few years of migration.^25^^–^^27^ In our study, this hypertensive effect was not observed for medium-term urban migrants. The initial increase due to rural-urban migration could have resulted from psychological stress induced by an unfamiliar fast-moving urban environment rather than as a result of changes in diet and physical activity.^6^^,^^28^ Furthermore, a recent review of stress and cardiovascular disease pointed to an array of mechanistic studies relating enhanced sympathetic nervous system activity to psychological stress and clinical hypertension.^29^ Psychological stress may attenuate as urban residents adapt. Indeed, attenuation of psychological distress among rural-urban migrants with increased length of stay in urban areas has been reported in China.^7^ Only young, recent urban migrants manifest hypertension in response to their migration, which may be associated with their inability to cope with stress.

Another mechanism of increasing hypertension due to urbanization may be related to changes in diet.^30^^,^^31^ Previous research suggested that urban residents had higher sodium consumption and greater sodium/potassium ratios than rural residents. This dietary effect may partly explain hypertension among long-term urban dwellers. As we did not observe an increased risk of four-year incidence among urban dwellers for middle- to old-age groups, dietary change related to sodium and potassium intake may not be a dominant factor linking urbanization with hypertension in our cohort. More research is needed to examine the level of sodium and potassium intake related to urbanization among Thais and comparable groups.

Compared with rural dwellers, rates of hyperlipidaemia were increased among anyone living in an urban area. Long-term urban dwellers (UUU) and urban migrants (RUU and RRU) all had higher risk of hyperlipidaemia. The risk of hyperlipidaemia increased rapidly after cohort members urbanized. Previous research has suggested that dietary changes can lower cholesterol levels within one year.^32^^,^^33^ Immigrants’ assimilation to an urban way of life (eg, changes in diet) after urbanization may raise cholesterol levels in a short period.^6^

Cohort studies are not usually designed to represent the general population, but they do provide sufficient heterogeneity of exposure to enable reliable estimates of relative risk based on internal comparisons.^34^ Selection bias when enrolling the cohort would not be expected to alter relative risk estimates, which are biological outcomes, when comparing exposed and unexposed individuals.^34^ ORs comparing groups within the cohort remain valid and can be generalized more broadly to the overall population.^34^^,^^35^

Of note, our study is based on self-reported measurements and would benefit from validation by reviewing medical records or physician reports. We validated self-reported hypertension by telephone interview and found results satisfactory. Since our cohort members live nationwide, validation studies based on physician reports or medical records would be very difficult. As we previously noted, any residual non-differential measurement error would move effect estimates towards a null result (a false negative), but in such a scenario, our non-null results remain valid.

We acknowledge that a higher OR of having hypertension or hyperlipidaemia for urban residents may be partly due to their higher chance of accessing health services. However, Thailand has had universal health care insurance since 2005. Thus, migrant Thais may actually have relatively equal access to health services in urban areas, unlike internal migrants in some other developing countries, such as China.^7^ Indeed, we include in the models two variables related to health service source and health insurance scheme. Thus, differences in use of medical care would not be expected to confound our assessments of the effects of urbanization on hypertension or hyperlipidaemia.

A central strength of this study is the large cohort size of over 50 000 adults who had been studied in 2005 and followed up in 2009. We examined outcome variables as both prevalence and incidence. Our national longitudinal data captured information on timing of rural-urban migration and the association with cardiovascular risks. Other studies of urbanization and cardiovascular risks in developing countries have been cross-sectional. These studies were either based in a single community with relatively small sample sizes or larger studies utilizing routinely updated national surveillance databases.^25^^,^^36^^,^^37^ These surveillance databases had limited information on health determinants.^18^^,^^36^ The TCS data, with a large sample size and a wide array of information on health behaviors across time, created a unique opportunity to examine temporal effects of urbanization on cardiovascular risks in developing countries.

### Conclusion

At the current rate of urbanization (1.6% per year), millions of Thais are at a higher risk of hypertension and hyperlipidaemia because they migrated recently. The risk of hypertension for young cohort members increased quickly after rural-urban migration. The risk of hyperlipidaemia also increased quickly after people migrated from rural to urban areas, particularly for those less than thirty years old and more than fifty years old—the youngest and the oldest birth groups in the TCS. Given the scale and expected continuation of urbanization as well as the grave consequences for cardiovascular health, we recommend developing an intervention plan to support and educate recent migrants when they arrive in Thai cities. The migration will continue for many years, and the results of this informative Thai cohort forewarn of consequences that occur quite early in the migration process, suggesting a narrow window of opportunity for intervention. The optimal design for such a novel intervention would itself require operational research, but Thai experience with major preventative programs is considerable.
